# Study of RNA Interference Targeting NET-1 Combination with Sorafenib for Hepatocellular Carcinoma Therapy *In Vitro* and *In Vivo*

**DOI:** 10.1155/2013/685150

**Published:** 2013-11-07

**Authors:** Song He, Ying-ze Wei, Gui-lan Wang, Yu-yin Xu, Jia-ming Zhou, Yi-xin Zhang, Li Chen

**Affiliations:** ^1^Department of Pathology, Nantong Tumor Hospital, Nantong 226361, China; ^2^Department of Pathological Anatomy, Nantong University, Nantong 226001, China

## Abstract

The aim of this study is to explore the inhibitory effects of RNA interference (RNAi) targeting NET-1 or combined with sorafenib on HCC *in vitro* and *in vivo* and the possible underlying mechanisms. The expressions of NET-1 mRNA and protein were detected by RT-QPCR and western blot. The ability of proliferation was determined by CCK-8 assay. Apoptosis was examined by flow cytometry (FCM). Abilities of migration and invasion were measured by scratch-wound assay and transwell assay. MHCC97H cells with stable transfection of NET-1shRNA were injected subcutaneously to prepare nude mice model of HCC and Caspase-3, Caspase-8, and Caspase-9 mRNAs of tumor tissues in different groups were examined. NET-1 mRNA and protein were reduced sharply in MHCC97H cells transfected with NET-1shRNA. The abilities of proliferation and migration were inhibited and apoptosis was promoted in either NET-1shRNA or sorafenib as compared with untreated cells *in vitro* and *in vivo* (*P* < 0.05). The mRNA levels of caspase-3, caspase-8, and caspase-9 of tumor tissues were reduced in different treatment groups compared with untreated group, particularly in combination group. (*P* < 0.05). The combination NET-1shRNA with sorafenib dramatically enhanced the effects of sorafenib antitumor ,which may involve in blocking ras signaling pathway and stimulating apoptotic pathways simultaneously.

## 1. Introduction

Hepatocellular carcinoma (HCC) is the third leading cause of cancer deaths worldwide [1], with some certain geographic regions in developing countries where the incidence of HCC is 16–32 times higher than in developed countries. Gene therapy, a new and promising therapeutic strategy, has been used for many cancers including HCC. SiRNA-targeted silencing of the genes associated with tumor cell proliferation or metastasis, as one method of gene therapy, shows great potency on HCC treatment. New EST tetraspanin-1, also called NET-1(C4.8, Tspan-1, P503S), a member of the tetraspan superfamily (TM4SF) [2–4], seems to be rather expressed in most HCC than in normal adult liver tissues [5]. This attractive characteristic of tumor-specific expression could made NET-1 as potential therapeutic target for HCC.

Sorafenib, an oral multikinase inhibitor approved by the US Food and Drug Administration for the treatment of patients with advanced renal cell carcinoma (RCC) and those with refractory HCC. Recent years, *in vivo* and *in vitro* studies have shown that sorafenib could inhibit tumor growth and disrupts tumor microvasculature through antiproliferative, antiangiogenic, and/or proapoptotic effects. Sorafenib represents an important advance in the treatment of advanced HCC and is the first systemic therapy shown to prolong survival in advanced HCC. A number of trials examining the combined use of sorafenib plus chemotherapy agents (e.g., fluorouracil [6], gemcitabine [7], or capecitabine plus oxaliplatin [8]) or of sorafenib plus other molecularly targeted therapies (e.g., sirolimus [9]) are currently underway and are yielding promising results. However, studies about gene therapy using RNAi technology combination with sorafenib on HCC rarely have been reported. Therefore in the present study, we used NET-1shRNA combined with sorafenib to explore a novel strategy for treating HCC *in vivo* and *in vitro*.

## 2. Methods

### 2.1. Cell Culture

Human HCC cell line MHCC97H was kindly provided by Liver Cancer Institute, Zhongshan Hospital, Shanghai. Cells were cultured with Dulbecco's modified Eagle's medium (DMEM) (Invitrogen, Carlsbad, CA) supplemented with 10% fetal bovine serum (FBS) (Invitrogen, Carlsbad, CA) in humidified atmosphere containing 5% CO_2_ at 37°C. The present experiments were divided into four groups including untreated group, sorafenib group, NET-1shRNA group and the combination NET-1shRNA with sorafenib group.

### 2.2. Transfection of Plasmids

pSilencer4.1-CMVneo-NET-1shRNA (NET-1shRNA) and pSilencer4.1-CMVneo-control shRNA (control shRNA) plasmids were designed and synthesized by Biomics Biotechnologies Co., Ltd. (Nantong, Jiangsu, China). One day prior transfection, cells were cultured in medium without serum and antibiotics. After mixed gently and incubated for 20 minutes at room temperature, the transfection mixture of shRNA and Lipofectamine 2000 (Invitrogen) was added into culture plates. After 6 h, the transfection mixture was replaced by DMEM supplemented with 10% FBS. Cells were harvested at 48 h after transfection. 

### 2.3. Real Time RT-QPCR

Total RNA was isolated with PicoPure RNA isolation Kit (Arcturus Bioscience, Mountain View, CA, USA) according to the manufacture's instructions. Glyceraldehyde-3-phosphate dehydrogenase (GAPDH) was amplified as an internal control [10]. The PCR primers were designed by Premier Primer 5.0 software (Table 1). Primers (20 pmol/*μ*L) were added into reaction buffer with a total volume of 25 *μ*L together with template RNA 4 *μ*L, Master Mix 12.5 *μ*L, and SYBR Green I 0.5 *μ*L. PCR was performed at the following conditions: 30 seconds at 94°C, 30 seconds at 62°C, and 40 seconds at 72°C for 50°C cycles. Real time RT-QPCR with SYBR Green PCR Master Mix (Applied Biosystems, Foster City, CA, USA) was performed using a MiQ machine (Bio-Rad Laboratories, Hercules, CA). The fluorescent signals were collected during the extension phase, Ct values of the sample were calculated, and NET-1 transcript levels were analyzed by 2-ΔΔCt method.

### 2.4. Western Blot Analysis

MHCC97H cells were harvested at 48 h after transfection. Cells were lysed with buffer containing 0.1 mol/L Tris-HCl (pH6.8), 4% SDS, 20% glycerine, 0.1% BPB, and 5% *β*-mercaptoethanol. The complex was heated in boiling water for 5 minutes. Proteins were separated by 10% polyacrylamide gel electrophoresis and then transferred onto polyvinylidene fluoride (PVDF) membranes (Millipore, USA) at 350 mA for 2 h, which was later soaked for 2 h on a blocking solution (Tris-buffered saline containing 5% nonfat dry milk and 0.01% vol/vol Tween-20), and incubated for one hour at room temperature in the presence of Anti-NET-1 rabbit polyclonal antibody [11, 12], or anti-GAPDH mouse monoclonal antibody (Sigma, USA) used as internal control, then incubated at 4°C overnight. After incubation, the membrane was washed 3 times, and peroxidase-conjugated goat anti-rabbit or goat anti-mouse secondary antibodies (ICN Laboratories, Irvine, CA; diluted 1 : 10,000) were added and incubated for an additional one hour. Reaction was visualized by the ECL chemiluminescence detection system (Pierce, USA) on radiographic films (Koda, USA). The molecular weight of NET-1 and GAPDH [13] were 76 kDa and 37 kDa, respectively. The results were analyzed using Image J software.

### 2.5. Cell Counting Kit-8 (CCK-8) Assays

#### 2.5.1. IC50 Determination

Sorafenib was purchased from Bayer Pharmaceutical Corporation, dissolved in sterile DMSO, and stored frozen under light-protected conditions at −20°C. Stock solutions of sorafenib were diluted in DMEM culture medium, and equal aliquots were added to individual wells so that the final concentrations were 0, 3, 9, 18, 30, 60, and 100 *μ*M. To determine the 50% inhibitory concentration (IC50) for sorafenib, the CCK-8 (Dojindo, Kumamoto, Japan) was used. MHCC97H cells were seeded at 5.0 × 10^3^ cells/well in 100 *μ*L of DMEM in 96-well microplates and incubated overnight at 37°C in a humidified atmosphere with 5% CO_2_. After the cultures were incubated for 0 h, 24 h, 48 h, and 72 h, we exchanged fresh DMEM gently and 10 *μ*L of CCK-8 solution was added to each well, and the plates were incubated for 2 h at 37°C. We measured the absorbance at 450 nm using an optical density (Microplate Reader 550; Bio-Rad, Tokyo, Japan) and calculated the IC50 concentration of sorafenib by the intersection of the plotted line (Figure 2). The IC50 of sorafenib was used in the growth, apoptosis, scratch-wound, and transwell assays.

#### 2.5.2. Growth Assays

5 × 10^3^ cells in each group were seeded in 96-well plates and cultured for 0, 24, 48, and 72 h, the optical density at 450 nm wavelength was measured through an automated plate reader and then cell growth curves were drawn.

### 2.6. Flow Cytometry (FCM)

Cellular apoptosis was determined using the Annexin V-FITC Apoptosis Detection Kit I (Clontech Laboratories Inc., USA) according to the manufacturer's protocol. Cells were seeded in 6-well plates at the density of 2 × 10^5^/mL and harvested by trypsinization then washed with cold PBS, centrifuged at 1000 rpm, resuspended in 400 *μ*L 1 × binding buffer, centrifuged again and removed supernatant. Cells were resuspended in 200 *μ*L 1 × binding buffer and transferred to a sterile FCM glass tube. 3 *μ*L Annexin V-FITC and 3 *μ*L propidium iodide were added and then incubated in the dark at room temperature. Cells were analyzed by FCM (FACSCalibur, Becton-Dickinson, USA) at 488 nm. The distribution of cells was analyzed using CellQuest software (Becton-Dickinson) in the FCM within 1 hour of staining. Data from 10,000 cells was collected for each data file. Apoptotic cells were identified as Annexin V-FITC-positive and P-negative cells. 

### 2.7. Scratch-Wound Assay

Cells were seeded at a density of 5 × 10^5^ in 24 well plates and incubated overnight. The day after, the surfaces of the dishes were mildly scratched with a yellow P200 pipette tip (Fisher) and images were taken under a Carl Zeiss Axiovert 25 (Thornwood New York, USA) inverted microscope with the use of a Cannon PowerShot G9 digital camera using a 40X objective, which were totally observed continuously for 72 h.

### 2.8. Transwell Assay

Uncoated or Matrigel-coated transwells containing 8 *μ*m pores were used for the assays (Costar, Corning, NY). Cells were seeded into the upper chamber in serum-free DMEM media. DMEM media containing 10% FBS was added to the lower chamber. Cells were fixed in 100% methanol 20 h later and stained with 0.2% concentration of crystal violet for 15 min at room temperature. Cells remaining on the upper side of the filter were removed with a cotton swab. The filters were then mounted onto cover slips and images were taken at 40X magnification. From these images, the number of migratory or invasive cells was counted.

### 2.9. G418 Selection

G418, an aminoglycoside antibiotic, is the most commonly antistable transfection reagents for screening in molecular genetic testing. Cells were seeded at the density of 2 × 10^5^ cells/well in 24-well plates and grew overnight. The medium was replaced with complete medium without FBS. NET-1shRNA and control shRNA were transfected into MHCC97H cells using Lipofectamine 2000 (Invitrogen) according to the manufacturer's instructions. The medium was replaced with a fresh medium of bovine serum (150 mL/L) after 6 h transfection. One day later, the transfected cells were selected by G418 (400 *μ*g/mL) (Huamei Biotechnology Company, Beijing, China) until positive clones were discovered after 14 days. The cells were cultured and finally selected by G418 (200 *μ*g/mL) for a further 14 days. Single clones were selected to build a stable transfected cell line.

### 2.10. Animals and Establishment of Tumor Model

#### 2.10.1. Animals

Nude BALB/c mice, female, 4–6 weeks old and weighing 18∼20 g, were obtained from Shanghai Slac Laboratory Animal (China) and housed under pathogen-free conditions according to the recommendations of NIH guidelines for care and use of laboratory. This study has been approved by the Animal Ethical Committee of Nantong University.

#### 2.10.2. Tumor Model

Nude mice were inoculated subcutaneously at the right anterior axilla with 1 × 10^7^ stably transfected NET-1 MHCC97H cells in 200 *μ*L PBS and at left anterior axilla with equal untreated cells, as autocontrol. The shortest axis (*a*) and the longest axis (*b*) of tumor were measured by caliper every day. The tumor volumes were calculated with the formula: volumes = *a* ^2^ × *b* × *π*/6. When the tumor volume reached 100 mm^3^ at least, the tumor-bearing nude mice model was established successfully.

The experiments composed of 12 nude mice, which were randomly assigned to four experimental groups. The four experimental groups were as follows: untreated MHCC97H cells group, stably transfected NET-1shRNA MHCC97H cells group, sorafenib treating MHCC97H cells group, and sorafenib treating stably transfected NET-1shRNA MHCC97H cells group. In sorafenib treatment group, the mice were given 100 mg/kg sorafenib in 100 *μ*L by peritoneal injection after tumor implantation established. All control mice received an equal volume of carrier solution by peritoneal injection.

At the 3 weeks of treatments, all treated mice were sacrificed, livers were excised and weighed, and one part was used for pathological examination and the others were stored at −80°C for detecting Caspase-3, Caspase-8, and Caspase-9 mRNA by real time RT-QPCR; the primer sequences were in Table 1. 

### 2.11. Statistical Analysis

All experiments were performed in triplicate and the results were expressed as mean ± standard errors. All data were analyzed with SPSS13.0 statistical software using student's *t*-test. Two-tailed *P* values of <0.05 were considered statistically significant.

## 3. Results

### 3.1. NET-1 Expression in MHCC97H Cells

**Figure 1:**
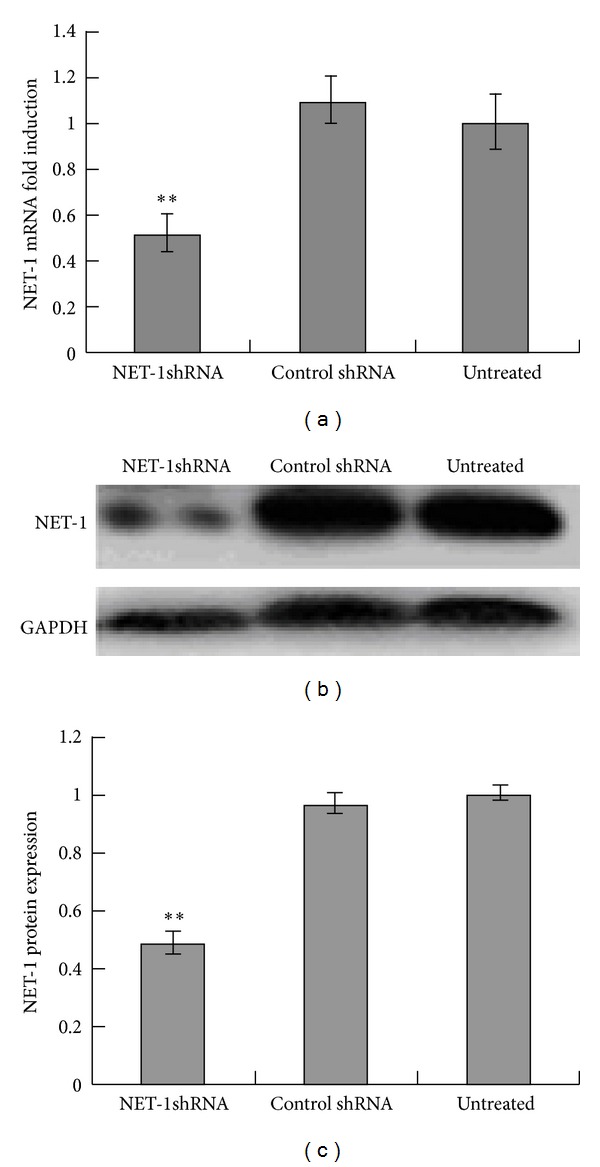
Inhibition of NET-1 mRNA and protein expression by NET-1shRNA in HCC cells line MHCC97H cells. (a) RT-QPCR showed NET-1shRNA resulted in 49% reduction of NET-1 mRNA levels, when compared with untreated group. (b) Western blot showed the intensities of NET-1 protein and GAPDH protein in these three groups. (c) Image J analyzed NET-1shRNA resulted in 51% reduction of NET-1 protein levels, when compared with untreated group (**P < 0.01).

RT-QPCR and western blot analysis was performed to determine whether transfection with NET-1shRNA resulted in a reduction of the expressions of NET-1 mRNA and protein. As compared with control shRNA, there were 49% and 51% reduction of NET-1 mRNA and protein levels in cells transfected with NET-1shRNA, and no significant reduction of NET-1 expression was found in cells transfected with either control shRNA or untreated group (Figure 1).

To evaluate the proliferation of MHCC97H cells treated by NET-1shRNA, sorafenib, and combination of them, cells growth curves were obtained through CCK-8 assay. There was a significant reduction of proliferation in all treated cells, and especially in combination NET-1shRNA with sorafenib as compared with untreated cells (each *P* < 0.01). However, there was no significant difference in cell proliferation rates between sorafenib and NET-1shRNA groups (Figure 3).

### 3.2. Proliferation of MHCC97H Cells

**Figure 2:**
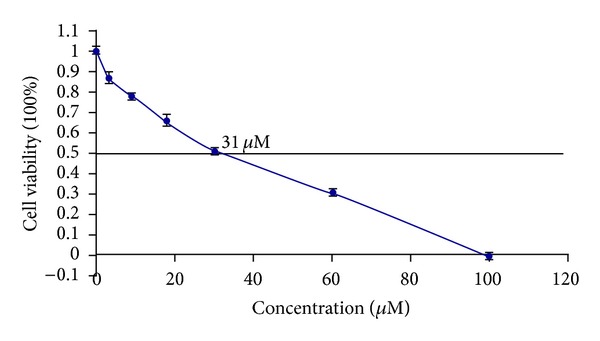
Dose-related cytotoxicity following 72 h exposured to sorafenib. The IC50 of sorafenib was indicated by the intersection of the plotted line. Sorafenib was 31 *μ*M.

**Figure 3:**
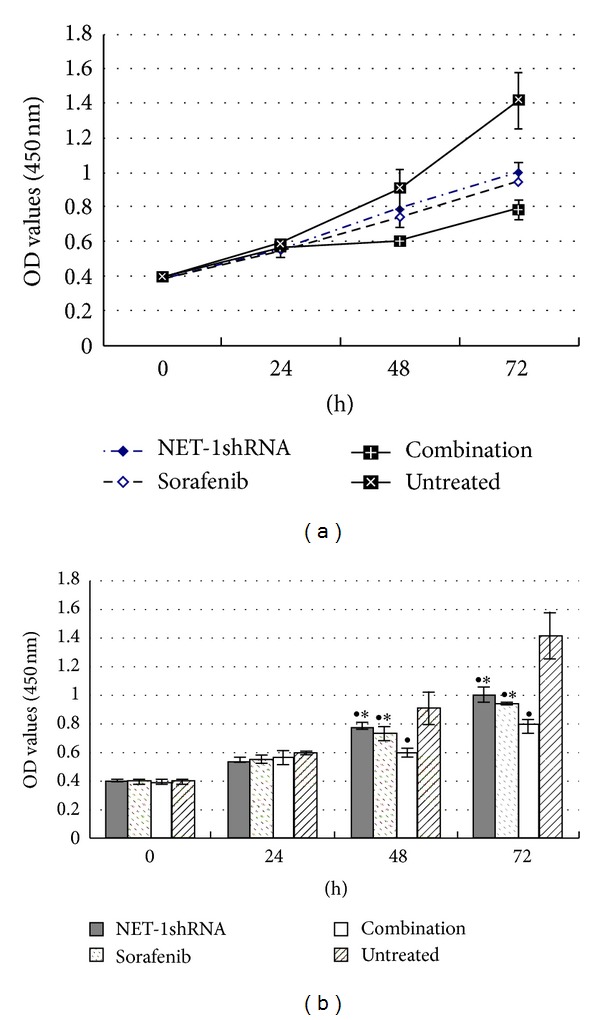
Growth curves showed the proliferation of MHCC97H cells until 72 h by CCK-8 assay. NET-1shRNA, sorafenib, and combination of NET-1shRNA with sorafenib resulted in 14.6%, 19.3%, and 33.3% reduction of cell proliferation at 48 h, in 46.5%, 50.6%, and 66.3% reduction of cell proliferation at 72 h. (^●^ *P* < 0.05 compared with untreated group. *P < 0.05 compared with combination group).

MHCC97H cells in 96-well culture plates were exposed to different concentrations of sorafenib for 48 h. IC50 of sorafenib was calculated as 31 *μ*M (Figure 2).

### 3.3. Apoptosis of MHCC97H Cells

**Figure 4:**
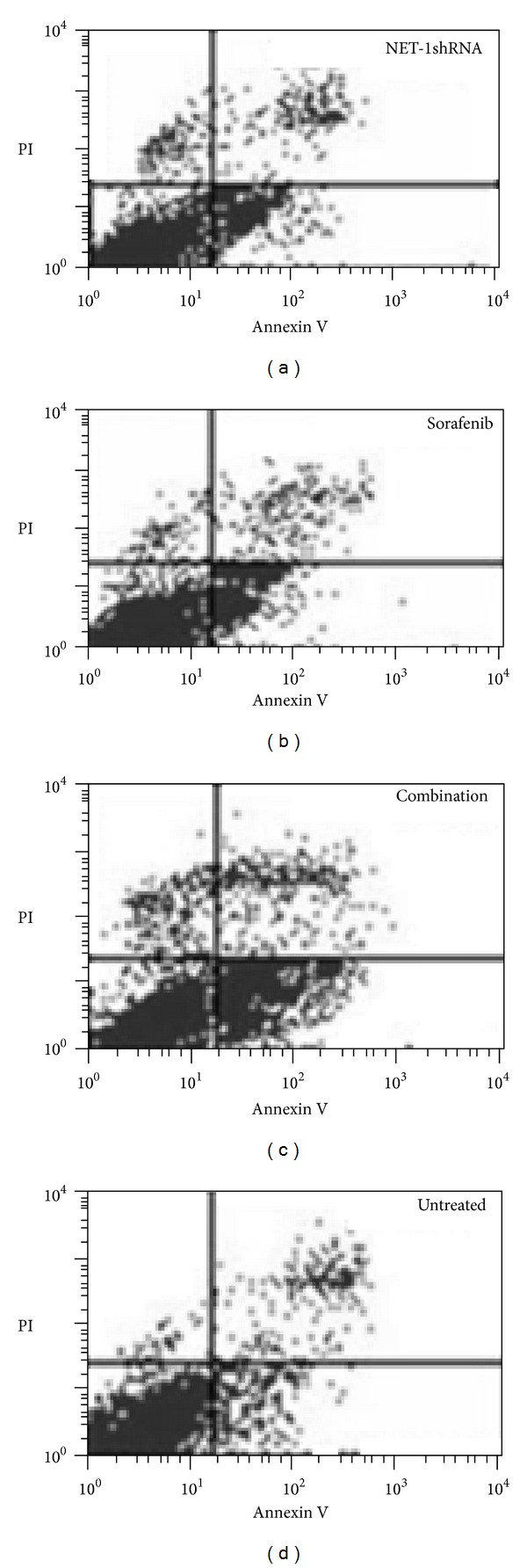
Percentage of apoptotic cells included both early- and late-stage apoptosis (AV+/PI− and AV+/PI+) was detected by FCM. The apoptosis rates of NET-1shRNA, sorafenib, combination, and untreated group were 24 ± 0.72%, 17.64 ± 0.46%, 51.34 ± 1.25%, and 6.3 ± 0.03%, respectively.

FCM was used to examine the apoptosis of MHCC97H cells. The apoptosis rate rose up in all three treated groups compared with untreated group. Interestingly, combination NET-1shRNA with sorafenib notably advanced the apoptosis rate than either sorafenib or NET-1shRNA group (*P* < 0.05, resp.). But there was no difference between these two single groups (Figure 4). 

### 3.4. Motility of MHCC97H Cells

**Figure 5:**
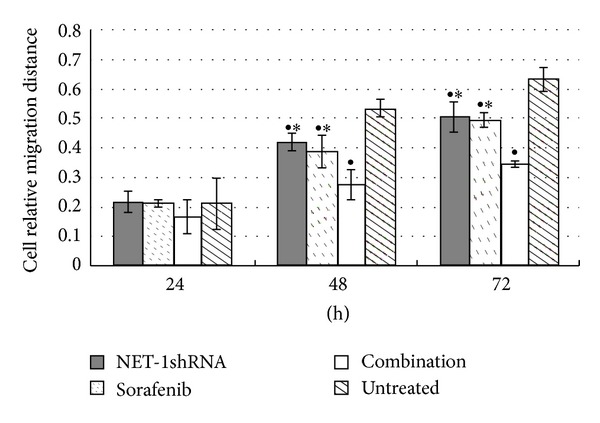
Cell relative migration distance at different time points in different groups. ^●^ *P* < 0.05 compared with untreated group. *P < 0.05 compared with combination group.

**Figure 6:**
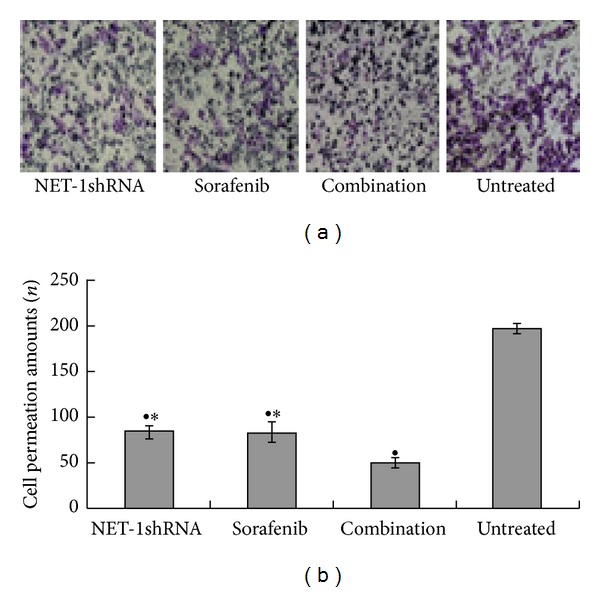
Motility of MHCC97H cells was inhibited by NET-1shRNA and sorafenib. (a) Trans-well chamber showed the permeation amounts of MHCC97H cells after 48 h of NET-1shRNA transfection or sorafenib treatment. (b) Trans-well chamber assay suggested the permeation amounts of MHCC97H cells in NET-1shRNA, sorafenib, and combination NET-1shRNA with sorafenib and untreated cells groups were 83.67 ± 6.03, 83.33 ± 10.01, 50.33 ± 4.73, and 197.00 ± 6.00, respectively. (^●^ represents *P* < 0.05 compared with untreated group. * represents *P* < 0.05 compared with combination group).

Scratch-wound and trans-well chamber assays were conducted to evaluate the migration of MHCC97H cells in the presence of sorafenib, NET-1shRNA, and both of them. Under these conditions, Scratch-wound (Figure 5) and trans-well chamber (Figure 6) showed MHCC97H cells display different migrating potential compared to untreated cells, respectively. Interestingly, there was no disparity between NET-1shRNA group and sorafenib group. Cells were observed under an inverted microscope.

### 3.5. Inhibition of HCC Model Growth by NET-1shRNA Alone or in Combination with Sorafenib

The tumor was used to mimic liver tumor growth in mice. The average tumor size presented statistical differences in these four groups. There was smaller tumor size in all treated groups than that in untreated group (*P* < 0.05, resp.). Notably, combination group in the inhibiting effect was superior to each single group (*P* < 0.05, resp.). However, no statistical difference was found between NET-1shRNA group and sorafenib group (Table 2).

**Figure 7:**
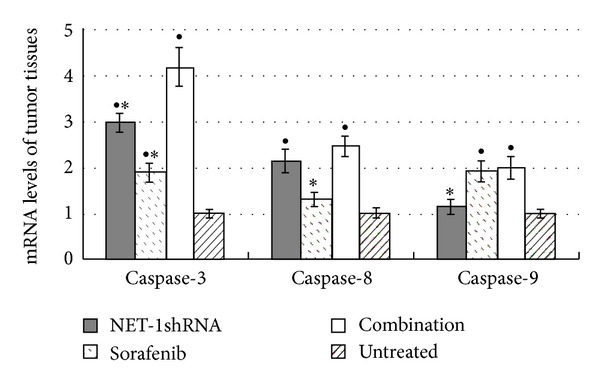
The expression mRNA levels of caspase 3, caspase 8, and caspase 9 of tumor tissues in NET-1shRNA, sorafenib, combination, and untreated groups. RT-QPCR showed that in NET-1shRNA, sorafenib, and combination groups caspase 3 mRNA levels increased by 2.99, 1.89, and 4.18 times, Caspase 8 mRNA levels increased by 2.14, 1.31 and 2.47 times, and Caspase 9 mRNA levels increased by 1.15, 1.91, and 1.99 times, compared with untreated group, respectively. (^●^ *P* < 0.05 compared with untreated group, *P < 0.05 compared with combination group).

To further investigate the mechanism about inhibiting tumor growth, we detected the mRNA levels of caspase-3, caspase-8, and caspase-9 of the tumor tissues from these four groups. The results of real time RT-QPCR showed caspase 3 in NET-1shRNA, sorafenib, and combination groups significantly increased, respectively, when compared with untreated group (each *P* < 0.05). Because Caspase 8 mRNA levels significantly increased in NET-1shRNA and combination groups, Caspase 9 mRNA levels significantly increased in sorafenib and combination groups, respectively, when compared with untreated group (each *P* < 0.05), so that increasing expressions of caspase-3, Caspase-8, and Caspase-9 mRNA in combination group were higher than that in each single group (*P* < 0.05, resp.) (Figure 7). 

## 4. Discussion

NET-1 as a novel tumor relative gene is over-expressed in many malignant tumors including the breast, uterine cervix, colon, esophagus, liver, lung, ovary, pancreas, prostate, gastrointestinal, and skin [10, 14–28]. Recent studies have shown that NET-1 is involved in a variety of processes such as oncogenesis, cell cycle control, apoptosis, and migration. Wollscheid et al. [17] found that NET-1 gene expression correlated to cell proliferation and may be used as a marker for cervical cancer prognosis.

RNAi technology is a powerful approach to silence mammalian gene expression for studies of gene function and has the potential for gene therapy. Synthetic siRNA can trigger RNAi response in mammalian cells and induce specific inhibition of gene expression. FDA approval has been granted for an investigational new drug license to test the use of expressed RNA sequences against HBV [29], bringing a promise for RNAi treatment on HCC. The specific shRNA used in our experiments has been exploited by Chen et al. and confirmed effective in previous research [10]. In the present study, NET-1 mRNA and protein expressions were significantly reduced by 49% and 51%, respectively, using NET-1shRNA.

NET-1 was proved to be associated with neoplastic cell proliferation [20]. Our data indicated an inhibition of tumor cell proliferation by 46.5% after single-agent sorafenib treatment, and 50.6% after NET-1shRNA transfection, respectively. Combined treatment with sorafenib plus NET-1shRNA significantly enhanced the antiproliferative effect of sorafenib on HCC *in vitro*. Furthermore, combined treatment also inhibited tumor volume of the tumor-bearing nude mice model more assertively than treated with a single agent *in vivo*. NET-1 belongs to the member of the guanine nucleotide exchange factor (GEF) family, the latter helps small G protein mutual transition from GDP to GTP, thus activating Ras and Rho. So our findings implied that NET-1 may promote tumor proliferation and growth through activating Ras signal transduction pathway, which mediates tumor cell proliferation, differentiation, and survival. The antitumor effect of sorafenib plus downregulation NET-1 by RNAi may achieve a synergistic effect on the inhibition of cell growth by inhibiting several relevant pathways. 

NET-1 has 30% identity with metastasis-associated tetraspans (e.g., Co-029 and Talla-1) [2]. Tetraspanins were implicated in regulation of signaling, motility, migration, and invasiveness [30]. Huang et al. showed a MK-induced NET-1 pathway contributing to migration/invasiveness of human head and neck squamous cell carcinoma cells then found RNAi silencing of NET-1 dramatically decreased MK-induced expression of MMP-2, which demonstrated the role of NET-1(TSPAN1) as one of various signaling components [31]. Our results revealed that migration and infiltration of MHCC97H cell were dramatically reduced after transfection of NET-1shRNA. Combination therapy with sorafenib and NET-1shRNA amplified the inhibition effects of sorafenib on tumor migration/invasiveness from 57.71% to 74.46%, compared with the untreated. Meanwhile, Sorafenib has been classified as a vascular endothelial growth factor receptor (VEGFR) inhibitor, which blocks tumor angiogenesis by decreasing microvessel density and circulating levels of VEGF. VEGF is one of the most potent angiogenic factors. MMP2 is a marker associated with angiogenesis as well as metastatic invasion [32]. Several studies demonstrated that the intensity of angiogenesis in HCC correlated with the risk of vascular invasion, metastasis, and patient prognosis [33, 34]. Therefore, these two agents (sorafenib and NET-1shRNA) in combination may boost the effect of each single agent.

Sorafenib has recently been found to induce apoptosis in several human cancer lines. Although the mechanism through which sorafenib induces apoptosis has not been fully elucidated, Yu et al. found sorafenib induced apoptosis by downregulating myeloid cell leukemia-1(Mcl-1) [35], which was presumed to be associated with the release of cytochrome c from mitochondria into the cytosol, caspase activation, and apoptotic cell death. *In vitro* apoptosis assay proved this hypothesis: the apoptosis rate of sorafenib treated group was higher than untreated cells. Moreover, our results revealed that caspase 3 and caspase 9 mRNA levels changed significantly after sorafenib treatment. Interestingly, from the results, we could find that caspase 3 mRNA level increased dramatically and the caspase 8 mRNA increased in NET-1shRNA and combination groups. The increase of caspase 8 mRNA led by NET-1shRNA perhaps hinting that NET-1 was probably an apoptosis related gene associated with the death receptor pathway mediated by membrane receptor. In addition, *in vitro* results demonstrated that reduction of NET-1 by RNAi may also induce apoptosis. Under our deduction, it is easy to explain why caspase 3 mRNA levels increased greatly in combination group. As for the relationship between NET-1 and caspase 8, we still need to explore the mechanism of NET-1shRNA induced apoptosis through other effective methods.

Taken together, in this study NET-1shRNA has obviously suppressed proliferation, survival, and migration/invasiveness of HCC both *in vitro* and *in vivo*. NET-1shRNA manifested inhibitory effect on HCC as well as sorafenib. Furthermore, combination of sorafenib plus NET-1shRNA showed better antitumor effect than single-agent treatment. These findings would bring a new therapy for HCC. 

## Figures and Tables

**Table 1:** Primer sequences of NET-1, caspase 3, caspase 8, caspase 9, and GAPDH.

Primer	Sequence	Product (bp)
NET-1-F′	5′-GTGGCTTCACCAACTATACG-3′	191
NET-1-R′	5′-GACTGCATTAGTTCGGATGT-3′	
Caspase-3-F′	5′-AGAACTGGACTGTGGCATTGAG-3′	191
Caspase-3-R′	5′-GCTTGTCGGCATACTGTTTCAG-3′	
Caspase-8-F′	5′-CATCCAGTCACTTTGCCAGA-3′	128
Caspase-8-R′	5′-GCATCTGTTTCCCCATGTTT-3′	
Caspase-9-F′	5′-TTCCCAGGTTTTGTTTCCTG-3′	143
Caspase-9-R′	5′-CCTTTCACCGAAACAGCATT-3′	
GAPDH-F′	5′-TGATGACATCAAGAAGGTGGTGAAG-3′	240
GAPDH-R′	5′-TCCTTGGAGGCCATGTGGGCCAT-3′	

**Table 2:** Comparing HCC growth treated by NET-1shRNA alone and in combination with sorafenib.

Groups	MHCC97H (cm^3^)
NET-1shRNA	1.354 ± 0.042^●★^
Sorafenib	1.201 ± 0.152^●★^
Combination	0.558 ± 0.018^●^
Untreated	3.459 ± 0.121
Empty vector	3.279 ± 0.084

The average tumor size in each group was 1.354 ± 0.042 cm^3^, 1.201 ± 0.152 cm^3^, 0.558 ± 0.018 cm^3^, and 3.459 ± 0.121 cm^3^, respectively, (^●^ *P* < 0.05 compared with untreated group. ^★^ *P* < 0.05 compared with combination group).
